# An Experimental Biomimetic Platform for Artificial Olfaction

**DOI:** 10.1371/journal.pone.0003139

**Published:** 2008-09-04

**Authors:** Corrado Di Natale, Eugenio Martinelli, Roberto Paolesse, Arnaldo D'Amico, Daniel Filippini, Ingemar Lundström

**Affiliations:** 1 Department of Electronic Engineering, University of Rome Tor Vergata, Rome, Italy; 2 Department of Chemical Science and Technology, University of Rome Tor Vergata, Rome, Italy; 3 Department of Physics, Chemistry and Biology, Linköping University, Linköping, Sweden; Instituto de Tecnologia Química e Biológica, Portugal

## Abstract

Artificial olfactory systems have been studied for the last two decades mainly from the point of view of the features of olfactory neuron receptor fields. Other fundamental olfaction properties have only been episodically considered in artificial systems. As a result, current artificial olfactory systems are mostly intended as instruments and are of poor benefit for biologists who may need tools to model and test olfactory models. Herewith, we show how a simple experimental approach can be used to account for several phenomena observed in olfaction.

An artificial epithelium is formed as a disordered distributed layer of broadly selective color indicators dispersed in a transparent polymer layer. The whole epithelium is probed with colored light, imaged with a digital camera and the olfactory response upon exposure to an odor is the change of the multispectral image. The pixels are treated as olfactory receptor neurons, whose optical properties are used to build a convergence classifier into a number of mathematically defined artificial glomeruli. A non-homogenous exposure of the test structure to the odours gives rise to a time and spatial dependence of the response of the different glomeruli strikingly similar to patterns observed in the olfactory bulb. The model seems to mimick both the formation of glomeruli, the zonal nature of olfactory epithelium, and the spatio-temporal signal patterns at the glomeruli level. This platform is able to provide a readily available test vehicle for chemists developing optical indicators for chemical sensing purposes and for biologists to test models of olfactory system organization.

## Introduction

Artificial olfactory systems have fascinated scientists for about 25 years, not least through the paper by Dodd and Persaud in 1982 [1]. They demonstrated that (four) chemical sensors with overlapping selectivity patterns could be used to discriminate among different odors. This discovery was based on the evidence that olfactory receptors are rather unselective; namely each receptor senses several kinds of odorant molecules and each odorant molecule is sensed by many receptors. The combinatorial signals pattern emerging form the ensemble of receptors is the key for odor classification, identification, and recognition [2]. This behavior surprisingly coincides with the properties of many solid-state chemical sensors, and since the eighties almost all the sensor technologies were used to assemble arrays of sensors with a certain capability to classify odors. The term electronic nose was coined to describe such sensor systems coupled with pattern recognition techniques, underlying in resemblance to human olfaction [3]. Although artificial olfaction was introduced with the purpose to provide artificial models of olfaction, the technological implementations of the concept were almost completely dedicated to the development of instruments rather than to make available a tool to biologists to test olfactory models. For this reason, progress in artificial olfaction systems was mainly focused on the development of artificial receptors. Receptor property is only one of the aspects of the rich structure of olfaction, while other important olfaction features, such as the large number of olfactory receptor neurons, their hierarchical organization, the odor patterns in the olfactory bulb, and the odor diffusion along the olfactory epithelium have been far less considered.

Among the possibilities to develop chemical sensors those based on optically active chemical indicators are extremely interesting for the possibility to synthesize a large variety of artificial receptor molecules whose chemical properties are modulated by molecular recognition events [4]. As an example, work by Suslick et.al. [5], and by ourselves [6] have shown the potential of ensembles of porphyrins and related compounds to correctly classify odorant molecules and odors. We recently demonstrated, using three colors excitation (a computer screen) and three colors detection (a digital camera), how optical fingerprints of the interaction between metalloporphyrins and volatile molecules can be used to identify and quantify various analytes [7].

In particular, optical sensors offer the possibility to extend the similarity between natural and artificial olfaction systems. To this regard Walt et al. have shown that the image formed by a bundle of optical fibers can easily allow the contemporaneous measurement of a large number of sensors when the tip of each fiber was properly chemically sensitized [8].

The properties of image sensors may be fully exploited imaging a chemically sensitive surface coated by a continuous layer of chemical indicators. In this situation, an image sensor provides a segmentation of the whole sensing layer in a number of elementary units corresponding to the pixel of the image. Eventually, since it is possible to evaluate the optical properties at the level of the single pixel, each pixel of the image corresponds to an individual sensor. To this regard, even low-resolution images may easily result in thousands of individual and independent sensing units.

This approach offers also the possibility to further develop artificial olfactory structures introducing a layer of glomeruli. In biological olfactory systems, both vertebrate and invertebrate, each olfactory receptor neuron (ORN) expresses only one of the genes proper of each species, and the signals of all the ORNs expressing the same gene converge to one glomerulus. Glomeruli are one of the most important units in the olfactory system where signals from receptor neurons of the same kind are elaborated for further coupling to higher levels in the brain [9], [10].

A kind of glomeruli layer was proposed by Walt et al. [11] that used self-encoded associations between individual artificial receptors, corresponding to the area of fiber tips as detected by the image sensor. In this system the association was determined according to the similar responses to a test gas and since the receptors are non selective, changes in the kind of test gas may result in a different arrangement. This feature mimics the functional arrangement of natural systems, where olfactory neurons with the same response properties and expressing the same receptor protein coalesc onto the same glomeruli [12], [13].

**Figure 1:**
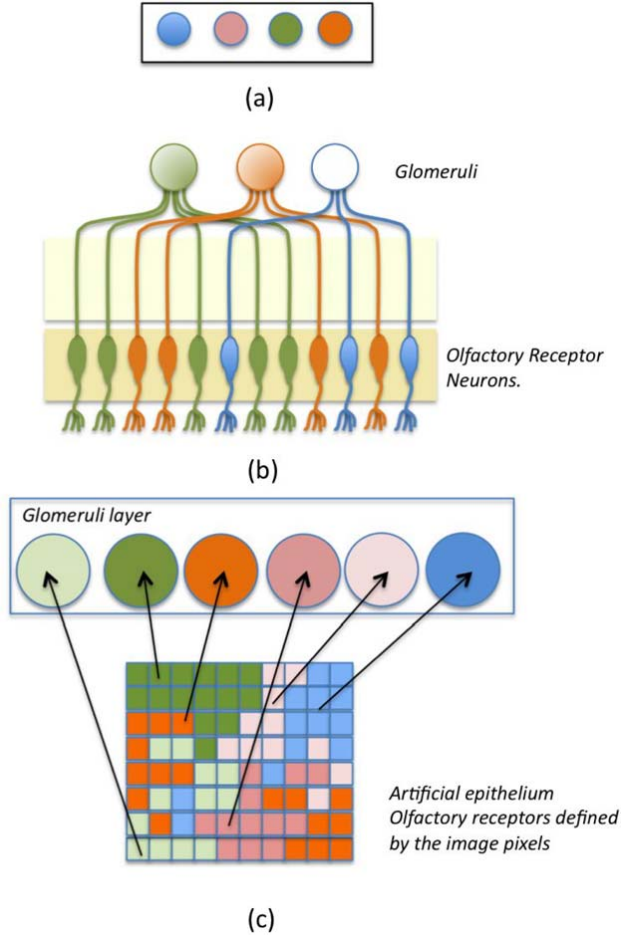
Comparison between standard sensor arrays as implemented in the state-of-the-art electronic noses, the scheme of the natural olfaction standard model and the architecture proposed in this paper. (A) Standard sensor arrays are formed by spatially controlled, homogenous, and separated spots of indicators. (B) In Nature, two layers are identified. The sensor level is formed by a spatially distributed layer of olfactory receptor neurons (ORN). Glomeruli form a second layer where the responses of homogeneous ORNs are evaluated, ORNs expressing the same receptor are connected to the same glomerulus. The application of the architecture of Nature to a standard sensor array is trivial and gives rise to coincident ORNs and glomeruli layers, which makes it difficult to mimick phenomena occurring in the olfactory epithelium with a standard sensor array. (C) In the system illustrated in this paper, ORNs are the pixels of the image of a sensing layer formed by an ensemble of spatially non-controlled, non-homogenous, and non-mixed indicators. The glomeruli layer is a mathematical object defined by the membership classes of the optical features of the different pixels. Similar to nature, a pixel (an ORN) belongs univocally to an artificial glomerulus regardless of its position in the sensing layer (epithelium).

In this paper we show that a collection of arbitrarily shaped regions of color indicators, like porphyrinoids, can be regarded as an artificial epithelium. Although not ordered, the porphyrin regions are not overlapping and then each pixel in an electronic image of the complete layer is characterized by only one porphyrinoid. In analogy to the fact that one ORN express only one receptor gene, each pixel of the image may be considered as an artificial receptor neuron and the output signal from such a neuron is the change in the optical properties of the pixel caused by the odor. The intrinsic optical properties of the pixels of the layer image are used to resemble the genetic manifestation of the membership of olfactory receptor neurons to different glomeruli, thus resembling the univocal association between an olfactory neuron and its glomerulus [12], [14]. In figure 1 a comparison between standard sensor arrays and the platform suggested by us is given. The resemblance of the proposed artificial olfaction architecture to the standard model of olfaction is also illustrated in figure 1.

Another important characteristic of natural olfaction is related to the spatial distribution of ORNs and to the fact that they are embedded in a diffusive media forming the so-called olfactory mucosa. When diffusing through the mucosa, odorant molecules are separated according to a gas-chromatographic principle [15]. To incorporate this property, the sensing molecules are dispersed in a polymer membrane that is sandwiched between two transparent sheets. One of the sheets is endowed with a hole allowing odorant molecules to freely diffuse through the polymer reaching the different porphyrinoid indicators.

This structure provides an artificial olfactory system characterized by a large number of individual ORNs (pixels) convergent to a layer of glomeruli. ORN-glomerulus association is driven by the optical characteristics of the particular porphyrinoid characterizing each pixel. It is important to remark that if each pixel is characterized by only one porphyrinoid (condition achieved if overlapping layers are avoided) the mapping of ORNs to the glomeruli layer is independent from the practical implementation of the sensing layer.

The diffusion of odorant molecules from one point of the artificial epithelium through the polymer matrix gives rise to spatio-temporal behavior of glomeruli signals that imitates the natural role of olfactory mucosa.

The structure has been tested in a simple experiment showing the different glomeruli signal patterns occurring as a consequence of the exposure to pure compounds. How the signals of individual ORNs are combined together to give rise to the glomerulus signal is still rather unknown. Single glomerulus signals are also influenced by intra- and inter- glomerular interactions [16], [17]. The platform proposed in our contribution provides a software model of glomeruli on which it is possible to apply any theoretical description of the glomeruli layer working principle. In this paper, this aspect will not be investigated, the simplest glomerulus transfer function is applied and the glomerulus signal is the instantaneous sum of the convergent ORNs signals. Even with this simple assumption, results are to large extent similar to what is found at the glomerular level in the natural olfactory system. It turns out that this experimental platform can provide a way to mimic both the zonal nature of the distribution of olfactory receptor neurons [18], [19] and the spatiotemporal response patterns observed in the olfactory bulb [17], [20].

## Results

**Figure 2:**
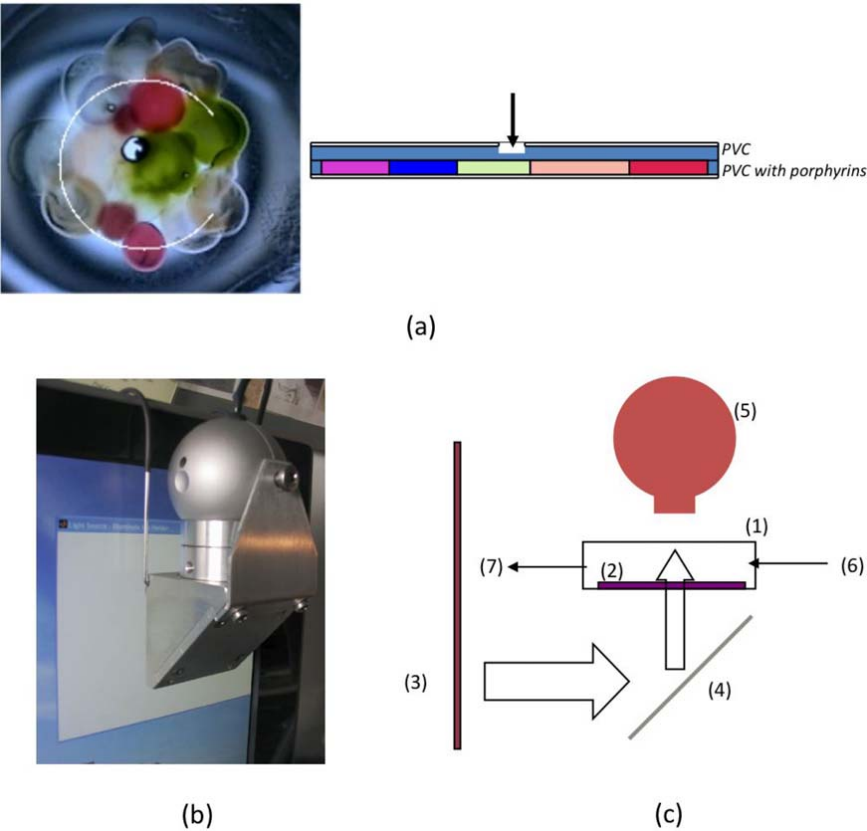
The artificial epithelium. (A) Image of the layer of porphyrinoids illuminated by white light, the (uncompleted) white circle indicates the region of the image considered in the analysis. The missing segment of the circle identifies the orientation of the artificial epithelium in other drawings. A schematic sideview of the artificial epithelium is shown to the right of the image. The epithelium is in contact with the odours via a hole in the upper plastic disc. (B) Experimental setup. (C) Schematic drawing of the measurement system: the artificial epitelium (2) is contained in a measurement cell (1) endowed with holes for gas inlet (6) and outlet (7). The transparent cell is illuminated, via a mirror (4), by an LCD computer screen (3) and the image of the artificial epithelium is captured by a standard computer camera (5).

The test structure was interrogated by a transmission mode optical arrangement where a standard digital camera recorded the image of the artificial epithelium illuminated by a computer screen. The exposure of the epithelium to the three color sequence creates a three colors optical fingerprint for each pixel of the image. To avoid errors due to image aberration only the pixels inside the white circle visible in Fig. 2a have been considered. For each pixel, the fingerprint depends both on the nature of the porphyrinoid and on its amount dispersed in the polymer volume imaged by the pixel. In the epithelium preparation each porphyrinoid was spotted in more than one place, varying the indicator as well as the polymeric solvent amounts. In order to maintain the univocal character of ORNs intermixing between the different indicator areas was avoided, but diffusion and drying effects created also zones of different optical densities.

**Figure 3:**
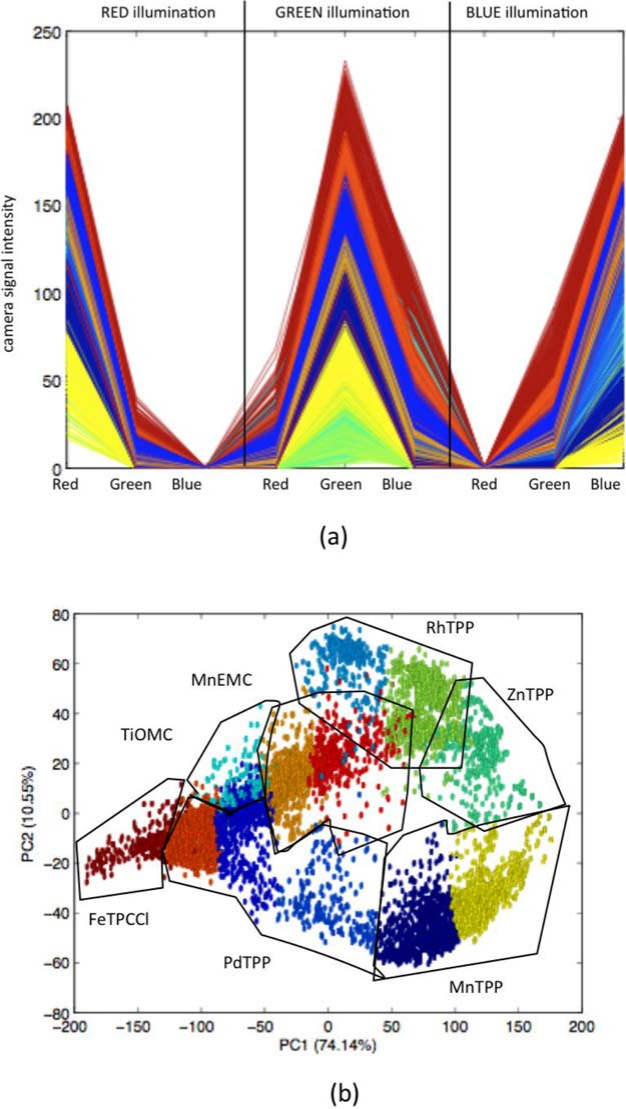
Fingerprints of the different pixels in the artificial epithelium. (A) Fingerprints extracted from the images of the artificial epithelium obtained in pure nitrogen with the setup in figure 2 Red, Green and Blue on the abscissas denote the intensities observed in the different camera channels. The lines are drawn just to amplify the visual differences between the optical fingerprints. (B) The fingerprints projected onto the plane formed by the first two principal components of a principal component analysis (about 84% of total variance represented). The plot illustrates the relationship between glomeruli and receptors (pixels), and it shows the scattering of the fingerprints due to concentration non-homogeneities of the color indicators used. As a consequence, the chosen classifier (k-means) requires a number of classes larger than the number of porphyrinoids in order to fulfill the principle that one glomerulus receives signals only from one kind of receptor (i.e. with similar intrinsic optical properties as defined by the k-means algorithm). Porphyrin acronyms are the same as used in the text.

Fig. 3a shows all the collected fingeprints. While in the case of a perfectly homogeneous chemical indicator distribution only seven different fingerprints should be visible in Fig. 3a, the obtained fingerprints form a continuous distribution, evidencing a high level of non homogeneity in the deposition. The relationship between non-homogeneity and intrinsic optical properties can be better studied considering the principal component analysis of the set of fingerprints. In figure 3b the Principal Component Analysis scores plot [21] of the fingerprints of all the considered pixels is shown. The plot illustrates the non-homogeneous distribution of porphyrinoids. It is worth to observe that in spite of the evident dispersion the individual character of indicators is preserved and then the classification of fingerprints into univocal classes is possible.

In this paper we consider each pixel of the image to be an individual ORN and its optical fingerprint the manifestation of the univocal nature of the kind of receptor characterizing the ORN. Then, the application of a classification algorithm to the set of fingerprints divides the thousands of pixels or artificial ORNs into a limited number of classes where ORNs carrying the same type of receptor converge. This collection of classes is a mathematical structure playing a role similar to that of the glomeruli layer found in natural olfaction.

In the simple classification scheme used in the present study, a k-means classifier [21], the number of glomeruli can actually be arbitrarily chosen. In this particular case, 12 glomeruli were chosen. This is the minimum number of glomeruli necessary to avoid ORNs misclassification, namely to avoid the convergence to the same glomerulus of ORNs carrying different kinds of receptors. It is worth to mention that misclassification, i.e. mixing in the same glomerulus the response of different receptors, tends to destroy the global specificity of the olfaction. Indeed, there is a natural trade off between discriminatory power and sensitivity (signal to noise ratio). A larger number of glomeruli increase the discriminatory possibilities whereas increasing the number of ORNs converging to the same glomerulus means a larger signal to noise ratio for a detected odour. Also in biological systems, a glomerulus connected to a large number of ORNs gives a larger sensitivity [22], [23].

**Figure 4:**
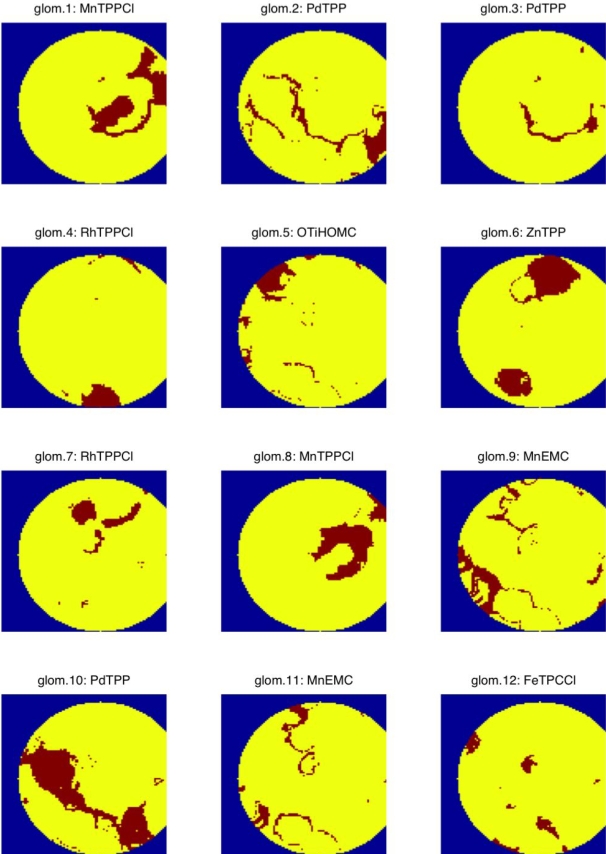
Segmentation of the artificial epithelium into glomeruli convergence areas. The intensities in the red, green and blue channel of the camera are measured at each illumination to give a fingerprint. Fingerprints are then classified in twelve groups (see figure 3). Due to the fabrication conditions of the polymeric layers in Figure 2, aggregation of porphyrinoids results in non regular patterns that are anyhow correctly captured by the procedure used to define the membership to a given artificial glomerulus. Some glomeruli indeed receive signals from defined and compact regions of the artificial epithelium while in other cases filamentary structures, likely due to diffusion of porphyrins inside the polymer matrix, are observed. The notation on the different images marks the glomerulus to which the specific area of the artificial epithelium delivers its signals. The indicator in the specific area is denoted as in the text.

Figure 4 shows the membership of the pixels (ORNs) of the artificial epithelium to 12 different glomeruli. It has been necessary to use more than seven glomeruli to fulfill the biological paradigm that one glomerulus receives signals only from one kind of ORNs. This is due to a non homogenous molecule density among the pixels carrying the same indicator, which means that not only the intrinsic adsorption spectra but also the total concentration of molecules enters into the classification. As a result, for some porphyrinods more than one glomerulus was defined. The result of the classification assigned one single glomerulus each to take care of the response of the layers of OTiHOMC, ZnTPP, and FeTPCCl; two glomeruli were assigned to MnTPPCl, RhTPPCl, and MnEMC, and finally three glomeruli were necessary to univocally take care of the response of the PdTPP layers. One fundamental property of a glomerulus is to improve the signal to noise ratio in comparison to that of a single olfactory neuron. This property applies also to the processing of optical sensors signals as elegantly shown by Walt et.al. [8] using optical fiber bundles. Figure 4 illustrates in a very direct way why our approach is more biomimicking than just using well defined spots of a number of given color indicators. The irregular distributions of the pixels contributing to the signal of one of the artificial glomeruli, which then serves the purpose of a well defined spot, i.e. the summary of pixels with similar properties, resemble much better the zonal- and distributed nature of ORNs in the olfactory epithelium. This distribution will be of main importance for the details of the spatio-temporal behavior of an artificial epithelium.

**Figure 5:**
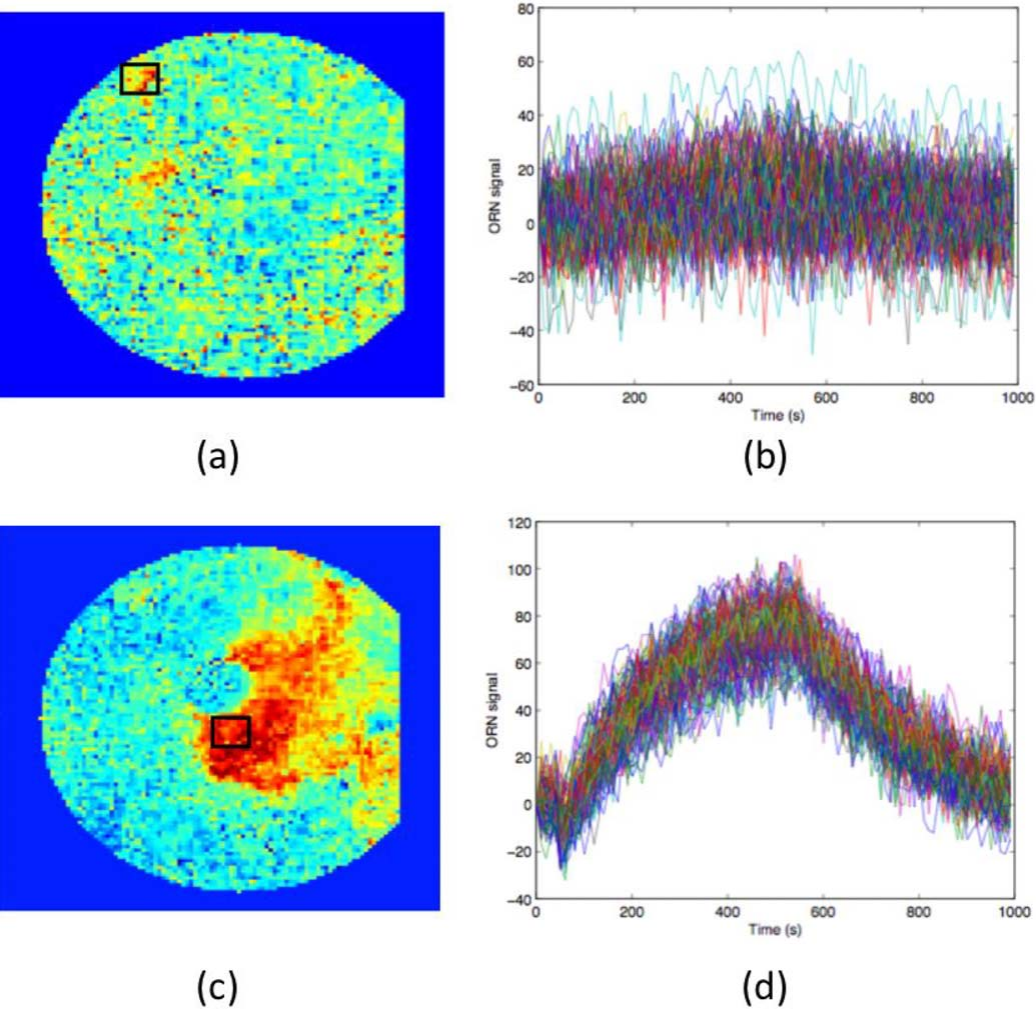
Example of signals from the artificial epithelium. (A) Color coded response image obtained as the difference between the image taken at the end of the exposure (50th image) to triethylamine and the first frame taken in the reference gas. The square marks a region where some of the most responding pixels are located. These pixels are related to one of the PdTPP layers. (B) Time evolution of the signals recorded from each pixel in the square marked in (A). The signals show the difference in optical response obtained, as described in the Materials and Methods section, upon the introduction and removal of triethylamine into/from the sample cell. Individual signals are immersed in noise and there is no evident response to the odour (triethylamine). (C) Color coded response image obtained as the difference between the 50th and the first image in case of exposure to butylamine. The square marks a region where some of the most responding pixels, related to MnTPPCl, are located. (D) Time evolution of the signals recorded from each pixel in the square marked in (C). The signals show the difference in optical response upon the introduction/ removal of butylamine into/from the sample cell. The difference was determined as described in the Materials and Method section. Also in case of a large and evident signal from individual pixels the noise is appreciable.

Figure 5 shows color coded results of the changes in the individual pixels of the artificial epithelium upon exposure to stimuli of triethylamine (Fig 5b) and butylamine (Fig. 5d). In case of butylamine the signal of a single pixel is large enough to be seen above the noise, while in the case of triethylamine the individual pixel signals do not emerge from the noise. In case of the response to butylamine it is also possible to appreciate the reversibility of the interaction.

The artificial glomeruli are modeled by software and any complex transfer function can be easily implemented both at inter- and intra- glomeruli level. Since a thorough description of such properties in the olfactory system is largely unveiled, the glomeruli are here described as nodes where the signals of all the ORNs (pixels) convergent to the same glomerulus are instantaneously summed.

**Figure 6:**
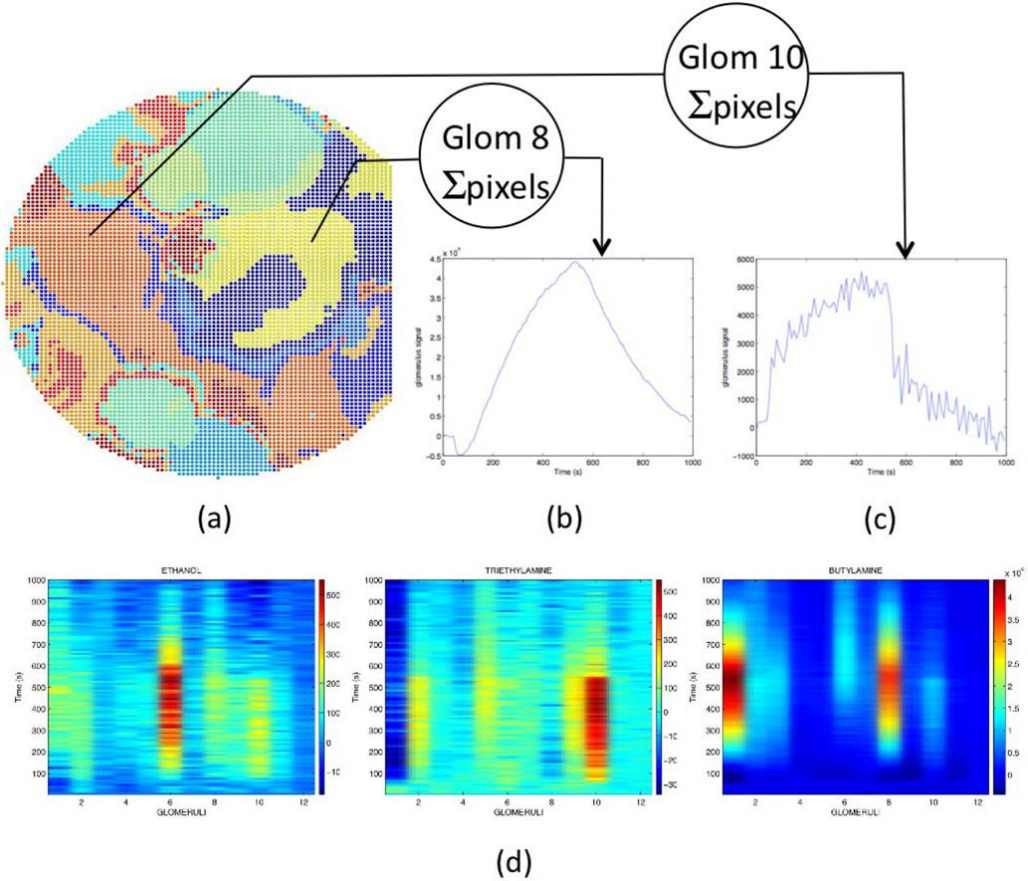
Receptor (pixel) convergence map on the different glomeruli and examples of the response of two glomeruli to two different odors. (A) Membership of each pixel to a given glomerulus (summary of figure 4). Different colors indicate the membership to different glomeruli. (B) Response of one of the glomeruli related to PdTPP during and after exposure to triethylamine. It contains as a part the pixels considered in Fig. 5b. A signal to noise ratio of 4 is obtained, which should be compared to that of individual pixels, not larger than 1.1. (C) Response of the glomerulus related to MnTPPCl during and after the exposure to butylamine. It contains as a part the pixels considered in Fig. 5d. The signal to noise ratio of individual pixels in this region is less than 7 while the glomerulus shows a signal to noise ratio of 90. (D) The responses of the twelve glomeruli to the three sample odors used (ethanol, triethylamine and butylamine) are shown as intensity coded color maps. Each odor gives rise to a different combination of glomeruli responses. (Different color scales for the three cases).

**Figure 7:**
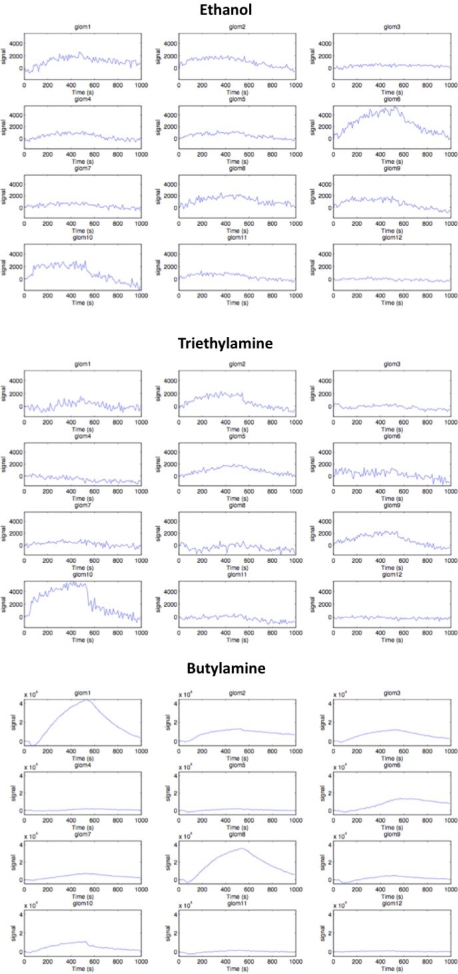
Time evolution of glomeruli signals. Normalized signals provided by the 12 glomeruli when the artificial epithelium is exposed to different odors illustrate the combinatorial response of the artificial olfaction platform and the reversibility of the interactions between odors and the artificial receptors (complementary to figure 6D).

The behavior of artificial glomeruli signals are illustrated in Fig. 6, where the time behavior of the responses at the glomeruli level (sum of the input signals) are shown. Figure 6a illustrates the individual ORNs (image pixels) whose different colors indicate the membership to a specific glomerulus (“summary of figure 4”). Color-coded time responses for all 12 artificial glomeruli are also shown for three different odours. The spatio-temporal results in Fig. 6d resemble similar plots for real glomeruli [20], besides the times involved, which are about 1000 times longer in our experimental setup. Figures 6b and 6c show the signals of the two glomeruli obtaining as a part the signals from the pixels (ORNs) inside the black rectangles in figure 5. As expected, in both cases, also for triethylamine, the signals at glomeruli level largely exceed the noise. The response of all glomeruli is detailed in figure 7. The figure illustrates also the reversibility of the interactions between odorants and porphyrinoids and the recovery of all glomeruli pristine signals.

For each gas a different set of glomeruli provides the maximum response. In case of ethanol, the largest signal is given by the glomerulus expressing the response of ZnTPP (glom 6) indicators with a lower contribution from MnTPPCl and some of the PdTPP glomeruli. In the case of triethylamine, the largest signal is provided by one of the glomerulus related to the PdTPP layers (glom 10) with minor responses from glomeruli expressing the responses of OTiHOMC and MnEMC. Finally for butylamine large responses from the glomeruli carrying the signals from MnTPPCl (glom 1 and glom 8) are observed, smaller responses are then provided by the glomerulus related to ZnTPP and one of the glomeruli related to PdTPP and RhTPPCl respectively.

Summarizing we find that the artificial epithelium provides a number of biomimicking features. First, it fulfils the rather self-evident condition that the added signal from many similar neurons is better than that from an individual neuron. There are, however, some additional properties that we like to stress. The different artificial olfactory neurons, i.e. the individual pixels, are distributed in irregular areas, similar to real olfactory receptor neurons. The experimental arrangement gives a diffusion limited temporal dependence to the response of a given artificial glomerulus, leading to a biomimicking spatiotemporal behavior of a collection of artificial glomeruli. The time scale is, however, not particularly close to the biological counterpart. The diffusion takes place in a circular geometry and without forced convection as in the nasal cavity. We believe that our experimental system may well be organized to better mimic also this aspect of olfaction including the separation properties observed in natural olfaction and shorter time constants. Another detail, which can be better mimicked, is the “mosaic” structure of the receptor distribution within a given zone of the olfactory epithelium, i.e. that receptors of different kinds are intermixed as illustrated in figure 1. This can be achieved e.g. by using a microspotter to (randomly) distribute spots of single pixel size (“single ORNs”) of the color indicators used.

## Discussion

The use of three-color excitation and detection based on a computer screen-web camera platform makes the demonstrated concept easily accessible for everyone interested. The experimental setup provides a large area multi-colour light source and three-colour images for further electronic manipulation. In the present paper we used the sum of the intensity changes in the three camera channels as the sensory signal. More information is available by using the intensity changes in each individual channel, separating absorption changes from emission changes. This can be used to create more artificial glomeruli from the same artificial epithelium using the intrinsic emission properties as the classification parameter and the change in emissions as the signals from the pixels as the artificial ORNs input to the glomeruli.

The use of the individual pixels in a digital image of the response to an odor as independent chemical sensors creates a simple way to obtain many individual sensors for signal evaluation in a biomimicking fashion. The chosen experimental system, where pixels with similar optical properties unsupervisedly converge into a layer of artificial glomeruli, is extremely simple. It provides several possibilities to vary the properties of the glomeruli, some of them already pointed out above. Different layouts and arrangements of the colour indicator spots are possible. The sample cell geometry as well as the choice of the polymer matrix for the colour indicators can be used to alter the influence of diffusion processes on the response to an odour. This degree of freedom was not used in the present study; the odour was supplied simply through a hole in the middle of the cover to the artificial epithelium with spontaneous diffusion as the only mechanism for exposure of the indicator areas. Through a more intelligent design of the sample cell pulsed operation or “sniffing” in geometry closer to that of the nasal cavity would be possible.

Although all these suggested improvements evidence the great potential of the suggested approach, it is also necessary to underline the important advantage of the simple to implement and simple to use experimental platform we showed here; the system is able to provide a readily available test vehicle for chemists developing optical indicators for chemical sensing purposes and for biologists to test models of olfactory system organization. This relates actually not only to the optical platform but also to the fabrication of the sensor array itself. Spontaneous formation of indicator spots of arbitrary sizes and shapes should be one of the simplest ways of making a sensor array for further evaluation. We have thus described a platform with a short distance between idea and experiment, which can be used both to model the importance of the distribution of ORNs and of diffusion in olfaction and for the development of electronic noses.

## Materials and Methods

For the test structure illustrated in this paper the following tetrapyrrolic macrocycles were used (the acronym used through the text are given in brackets): (5,10,15,20-tetraphenylporphyrin)zinc [ZnTPP], (5,10,15,20-tetraphenylporphyrin)palladium [PdTPP], (5,10,15,20-tetraphenylporphyrin)manganese chloride [MnTPPCl], (5,10,15,20-tetraphenylporphyrin)rhodium chloride [RhTPPCl], (5,10,15-triphenylcorrole)iron chloride [FeTPCCl], (2,3,8,12,17,18-hexamethyl,7,13-dimethylcorrole)manganese [MnEMC] and (2,3,7,8,12,13,17,18-octamethylcorrole) oxotitanium [OTiHOMC]. The artificial epithelium was prepared placing drops of a tetrahydrofuran solution of a PVC membrane containing the tetrapyrrole indicator (membrane composition in weight: tetrapyrrole/PVC/bis(2-ethylhexyl)sebacate 1∶33∶66) onto a 25 mm diameter Thermanox® plastic coverslip (Nunc™). The sensing film was overlaid with an empty PVC membrane and then covered with another plastic coverslip. The upper cover slip was endowed with a hole to expose the polymeric film to odor and to allow odor molecules to freely diffuse into the film and interact with the indicator molecules. The appearance of the epithelium is visible in Fig. 2a. As partially evident in this figure, the chosen porphyrinoids are characterized by well separated colors (or optical spectra). The experimental set-up is shown in figure 2b and 2c: the artificial epithelium was enclosed in a sample cell with transparent walls and endowed with inlet and outlet for odour delivery. The cell was placed in an optical path connecting a standard LCD monitor (Philips 170S4) with a computer camera (Philips SPC650NC/97). This seemingly trivial instrumentation allows optical fingerprints of color indicators and their interaction with guest molecules to be determined, where the fingerprints contain information about both absorption and fluorescence [7], [24].

The optical properties of the artificial epithelium were fingerprinted with a three colors sequence, pure red, pure green and pure blue, rgb = (255, 000, 000), (000, 255, 000) and (000, 000, 255) respectively. For each color the camera recorded the intensity received in its three channels (red, green and blue). A total of 9 values thus define the optical fingerprint of each pixel (Figure 3a). Fingerprints have been classified with a k-means, an unsupervised algorithm providing the membership of each pixel to a glomerulus [21].

The response to odors was measured in experiments where the artificial epithelium was exposed to vapors of ethanol, triethylamine, and butylamine at a concentration given by the saturated vapor pressure at room temperature diluted five times in a nitrogen flow (200 sccm total flow). Each experiment lasted 1000 s and a measurement point was taken every 10 s. The epithelium was exposed to gas from measurement 1 to measurement 50 and then left exposed only to nitrogen. Measurement 1 (in carrier gas only) was used as the reference, see below.

The response was evaluated illuminating the epithelium with the three pure colors. For each color the camera response in the three channels was acquired. It resulted in nine measurements of which only six were considered meaningful. The camera channel corresponding to the illuminating color (color- camera filter: red-red, green-green and blue-blue) provides information about absorption and the camera channels corresponding to longer wavelengths than the illumination (green-red, blue-green, and blue-red) contain information about emission. The six signals were simply summed to form the individual ORN signal. In order to evaluate only the changes induced by the exposure to odors in each experiment the signals recorded in the first measurement were subtracted from all of those following.
